# Incidence and predictors of groin complications early after coronary artery intervention: a prospective observational study

**DOI:** 10.1186/s12912-019-0349-8

**Published:** 2019-06-28

**Authors:** Maan Sh. AL-Momani, Mohannad Eid AbuRuz

**Affiliations:** 1 Royal Medical Services, Amman, Jordan; 2 Applied Science Private University, Po box 142 Shafa Badran, Amman, 11934 Jordan

**Keywords:** Groin complication, Percutaneous coronary intervention, Femoral access site, Coronary artery disease, And Jordan

## Abstract

**Background:**

Coronary artery disease remains the most common single cause of death worldwide. Percutaneous coronary intervention is an appropriate management for coronary artery disease which is not free from its potential complications. The purpose of this study was to determine the incidence rate and the predictors of groin complications post percutaneous coronary intervention in cardiac catheterization laboratories in Jordan.

**Methods:**

This was a prospective observational study with a consecutive sample of 300 patients post percutaneous coronary intervention procedure. Data were collected from the cardiac health care center using a pre-structured observational sheet. Any groin complication developed within the first 24 h post procedure was recorded. All correlated variables were analyzed using logistic regression.

**Results:**

The sample included 237 (79%) men and 63 (21%) women with a mean age of 57.46 ± 10.51 years. A total of 114 patients (38%) developed one or more groin complications. Ecchymosis was the most frequent groin complication; 102 (34%). Females and participants greater than 65 years were nearly two times more likely to develop groin complications (OR = 2.13, *P* = .024, 95% CI: 1.11–4.01) and (OR = 2.14, *P* = .023, 95% CI: 1.11–4.13) compared to other groups. Patients with a systolic blood pressure before sheath removal greater than 180 mmHg were about ten times more likely to develop groin complications (OR = 9.82, *P* = .001, 95% CI: 2.58–37.37).

**Conclusions:**

Different factors can increase the risk of groin complications post percutaneous coronary intervention. Therefore, identification of high risk groups (i.e. females) might help in the application of different methods to control these complications.

## Background

Cardiovascular disease (CVD) is a group of disorders of the heart and blood vessels that includes coronary heart disease, cerebrovascular disease, peripheral arterial disease, congenital heart disease, deep vein thrombosis and pulmonary embolism [1]. Cardiovascular disease is the leading cause of death among adults worldwide [1–4]. Cardiovascular disease accounted for more than 17.3 million deaths in 2013, a number that is predicted to grow to more than 23.6 million by 2030 [1]. Cardiovascular disease represented 31% of all global deaths in 2013, and it affected males and females almost equally [1].

Coronary Artery Disease (CAD) is the most common form of CVD [1–4], and is the most common single cause of death worldwide [1–4]. Deaths related to CAD increased by an estimated 41.7% from 1990 to 2013 [1]. Coronary artery disease is responsible for 45.1% of all deaths in the United States [1], and 39% in Europe [4]. Furthermore CAD was responsible for 294 per 100,000 of all deaths in the Middle East, and 18% of all deaths in Jordan [2, 5].

Coronary artery disease has negative consequences on the patient’s physiological, economical, social, and psychological status [6]. Moreover, it affects the patient’s quality of life, spiritual domains, and activities of daily living [6]. Therefore, accurate management for CAD is necessary. The significant developments in the diagnosis and treatment of CAD made Percutaneous Coronary Intervention (PCI) an appropriate treatment of CAD [7, 8].

Even though this procedure has reduced morbidity and mortality rate for cardiovascular disease, it does have its own complications [9]. Major complications post PCI occur infrequently in about 3% of all procedures [9], including but are not limited to death, myocardial infarction, stroke, and emergency bypass surgery [9]. On the other hand, the minor complications happen more frequently and they include renal insufficiency (8%), anaphylactic reaction to contrast (0.23%), arrhythmias (0.84%), infection (0.64%) and vascular complications (0.1 to 61%) [10–13]. The vascular complications related to access site (groin area in this study) are the most frequent complications [10, 12, 13].

The most frequent groin complications are: a) major complications (bleeding, large hematoma, pseudoaneurysm, retroperitoneal bleeding, arterial occlusion, and arteriovenous fistula), and b) minor complications (oozing, small hematoma, and bruising (ecchymosis)) [10, 14–18]. The incidence rates of these complications vary across studies; the lowest rate mentioned was 0.1% and the highest rate mentioned was 61% [10, 16, 17]. This wide range is probably raised from methodological variations between studies.

Nearly all conditions and requirements of the PCI procedure may contribute to the development of vascular access complications. These conditions include: a) the techniques used by the interventional cardiologist, such as localization of the access site and the number of punctures (at time of femoral cannulation), the dilator sheath size, time of sheath removal, and the procedure time [10, 11, 19–23], b) administration of different kinds of anticoagulants and antiplatelets medications, c) using different methods to achieve homeostasis at the access site, and d) the nursing care [9, 10].

The socio-demographic and clinical characteristics were documented in the literature to be risk factors for groin complications development post cardiac catheterization procedure (CCP). Advanced age was mentioned as a factor associated with the development of some forms of groin complications (e.g ecchymosis, hematoma) [18, 24]. Moreover, female gender was also reported to be a risk factor for bleeding complications post CCP [25]. Furthermore, body mass index (BMI) (extremely under-weight, or obese) has been identified as a correlated factor with hematoma development [24], and femoral artery pseudoaneurysm (FAP) development post CCP [26].

Clinical characteristics such as history of hypertension, history of diabetes mellitus, peripheral vascular disease, and renal failure were concluded as associated factors with groin complications development post CCP [6, 11, 26, 27]. The hypertension was reported as risk factor for FAP development post CCP [26, 27]. In addition, the renal dysfunction was associated factor with FAP development post CCP [27]. Further, patients who had type one diabetes mellitus were prone to groin complications development post CCP [14].

Limited studies were conducted in developing countries including Jordan about the predictors of groin complications post PCI. Only two studies were done in Jordan for this purpose. The first study was conducted to recognize the incidence time and predictors of hematoma development after manual compression to the access sites after diagnostic or interventional PCI procedures [24]. The study found that advanced age, obesity, systolic blood pressure more than 160 mmHg, and anticoagulants were significant predictors for hematoma development. Whereas comorbidities diabetes mellitus, hypertension, peripheral vascular disease, bleeding disorders, and gender were not significant predictors for hematoma development [24]. The second study concluded that gender, age, hypertension, and diabetes mellitus were found to be not significant variables as risk factors of vascular complications [28]. Therefore, the purpose of this study was to determine the incidence rate and the predictors of groin complications post PCI procedure in primary cardiac catheterization laboratories services in Jordan.

## Methods

### Research questions

1) What is the incidence rate of groin complications post PCI procedure in primary cardiac catheterization laboratories services?, 2) Is there an association between socio-demographic characteristics (age, gender, BMI, diabetes mellitus, renal failure, and hypertension) and groin complications post PCI procedure?, and 3) What are the most common predictors of groin complications post PCI procedure?

### Design, sample and sampling

A prospective observational correlational design was used to address the study purpose. A consecutive sampling technique was used to allocate participants. The sample involved all PCI patients who were hospitalized in Cardiac Health Care Center (CHCC) for elective PCI procedure and met the eligibility criteria from February 2018 until March 2018.The inclusion criteria comprised all PCI patients aged more than 18 years with femoral access and agreed to participate in the study. The exclusion criteria were: a) patients with unstable hemodynamic status; hemodynamically unstable patients need to keep the arterial sheath in extra time as an arterial line for invasive blood pressure monitoring, and for obtaining gases sample, b) non PCI procedures; as they underwent different conditions and anticoagulant doses than PCI procedures, c) patients who underwent through any catheterization procedure (through femoral approach) within a 1 month duration; those patients may have groin complications before PCI procedure, and d) patients who were unable to consent due to existing psychiatric conditions.

**Fig. 1:**
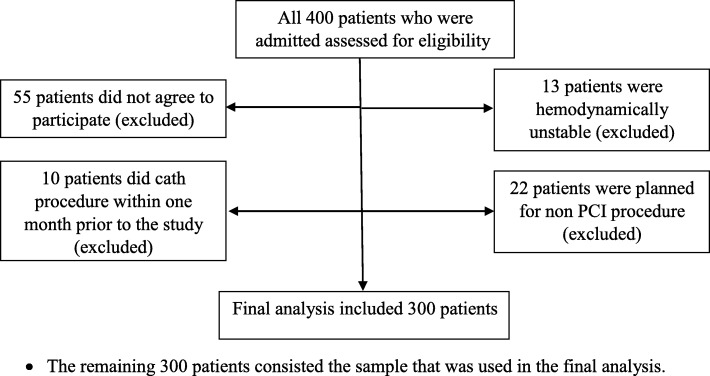
Patient flow diagram

A power analysis was conducted to determine the required sample size for this study. The estimated sample size was done using the G* power 3.1.9.2 software for logistic regression. In order to be conservative in estimating the effect size, a medium effect size of (0.3) for logistic regression was considered. Other assumptions included logistic regression as a major statistical test with an estimated OR of 2.33, 70% male gender, an alpha of 0.05, two tailed test, and a power of 0.80. In order to make the sample more representative, to compensate for any attrition that might occur during the study, and to adjust for the nature of the statistical test used; about 30% of the calculated sample size was added to the total number of the estimated size of the sample. Based on these assumptions, the overall sample size needed was 300 participants. The recruitment process was continued till this number was reached, Fig. 1.

### Settings

The study was conducted in CHCC in Amman. Cardiac Health Care Center is a specialized health care center for all heart diseases and surgeries. The CHCC covers a wide variety of patients, as it receives patients from both the public and private health sectors in Jordan, in addition to the military sector. It has a capacity of 215 beds, 5 cardiac catheterization laboratories, and 6 theaters for all kinds of cardiac surgeries. The CHCC provides health care for all cardiac disease patients in Jordan and includes patients from other countries. In CHCC all types of PCI procedures are performed. For instance, in 2017, 3651 PCI procedures were performed. The center has 20 cardiologists and 10 fellows among which one cardiologist and 2 follows are presented 24 h a day. Moreover, the center has 35 cath-lab technicians and approximelty 400 nurses with at least Bachelor degree. According to the CHCC policy, all PCI patients should stay for at least 24 h after the procedure.

### Measurement of variables

An observation sheet developed by the researchers was used to collect data. The items included in the observational sheet were adopted from the reviewed literature. Moreover, the accuracy and relevancy of the instrument was assessed and evaluated by a panel of experts. The Observation sheet included four parts: the first part consisted of demographic characteristics and comorbidities, the second part consisted of the procedure conditions, the third part consisted of the arterial sheath removal conditions, the fourth part consisted of the suspected groin complications.

Demographic characteristics sheet included: age, gender, height, weight and body mass index (BMI). Comorbidities include medical history of diabetes mellitus, hypertension, peripheral vascular disease and renal failure. The procedure conditions sheet included: procedure starting time, heart rate (HR), site of puncture (right, left), number of punctures, procedure performed, sheath size: (5F, 6F, 7F or 8F), blood pressure when the procedure started and when it ended, anticoagulant and dose used, use of glycoprotein IIb/IIIa inhibitors (Aggrastat), procedure duration, activating clotting time (ACT) at the end of the procedure, amount of dye used, time of procedure, fluoroscopy time and fluoroscopy dose.

The arterial sheath removal conditions sheet included: ACT result, sheath removal time, heart rate, blood pressure before and after sheath removal, hemostasis time (compression duration), and presence of dorsalis pedis pulse or posterior tibial pulse prior and after sheath removal.

The groin complications sheet included the complications which were assessed whether present are: bleeding, hematoma (large or small), oozing, ecchymosis, arteriovenous fistula (AVF), retroperitoneal hemorrhage, pseudoaneurysm, and femoral occlusion (thrombosis). These complications were monitored at four points of time; before sheath removal, after compression completion, six hours after sheath removal, and 24 h after sheath removal. The number of complications was recorded if present. Study variables and groin complications were considered and identified operationally as in Table 1.

**Table 1:** Operational definition of study variables

Variable	Definition
Hypertension	The categories of blood pressure (category: systolic mmHg(S), diastolic mmHg(D)) as: optimal (S < 120, D < 80), normal (S < 130, D < 85), high normal (S 130–139, D 85–89), stage1(mild) (S 140–159, D 90–99), stage 2 (moderate) (S 160–179, D 100–109), and stage 3 (severe) (S ≥ 180, D ≥ 110) [29].
Body mass index	Weight in kilograms divided on height in meters squared (kg/m2). Categories as: underweight (Less than18.5 kg/m2), normal (Between 18.5 kg/m2 and 24.9 kg/m2), and overweight (More than 25 kg/m2) [29].
Number of femoral punctures	The number of groin punctures to achieve proper femoral artery cannulation.
Oozing	Presence of any leakage of blood from the puncture site. The blood tainted area is < 3 × 3 cm^2^ on the gauze, but it does not reach the level of bleeding [18].
Ecchymosis (bruising)	Presence of any skin discoloration associated with pain and minor swelling [16].
Bleeding	Considered present when there is more than 2 g/dl loss from baseline of hemoglobin from puncture site [18].
Hematoma	Swelling mass surrounding the puncture site hard, palpable, and tender (Small hematoma: < 5 cm in diameter, Large hematoma: > 5 cm in diameter) [17].
Pseudoaneurysm	Pulsatile mass; presence of a palpable mass with corresponding movement to systole and diastole diagnosed by Doppler ultrasound detecting flow [14].
Retroperitoneal bleeding	Moderate to severe back pain associated with hypotension and tachycardia diagnosed by computed tomography [17].
Femoral occlusion (Thrombosis)	Complete occlusion of the femoral vessel as detected by the absence of distal foot pulses, and confirmed by Doppler ultrasound detecting flow [17].
Arteriovenous fistula	Abnormal communication between the femoral artery and vein produces palpable thrill and bruit sound confirmed by a stethoscope placed over the puncture site, and by Doppler ultrasound detecting flow [17].

### Ethical consideration

Prior to data collection, approval was sought from the Institutional Review Board from both Applied Science Private University and CHCC to protect the participants’ rights. Then, after screening patients for the eligibility criteria, they were asked to participate in the study. The patients who accepted to participate in the study (after providing a detailed explanation about the purpose, benefits and risks, and duration of the study) signed an informed consent form. It was also explained that they can withdraw at any time they want to. After signing the informed consent form and explaining the purpose of the study, the observational sheet was coded numerically to maintain confidentiality. Data were kept in a locked cabinet for protection to ensure the confidentiality of the information with access only to the principal investigator and the co-investigator.

### Data collection procedure

Data were collected using the observational sheet that was developed by the researcher prospectively at four points of time. The affected side was assessed immediately after PCI procedure completion, at sheath removal time, after 6 h of sheath removal, and after 24 h. The data were collected by the researcher himself because he is working at the site. Data were verified by the cardiologist in the unit who was available 24 h a day as per center policy. There was an agreement on the complications between the researcher and the cardiologist based on the operational definition. The same types of medical measurement tools were used; electronic sphygmomanometer, electronic body weight device, stadiometer (height measure tool), and a flexible ruler. All instruments were calibrated to ensure the consistency and validity of the obtained readings, and to reduce any possible bias that could occur from the instruments [30].

### Data analysis

Data were analyzed using the statistical package for social sciences (SPSS) IBM Corporation, New York, USA version 21. Descriptive statistics were conducted to analyze the following demographic variables: gender, age, BMI, and comorbidities (diabetes mellitus, hypertension, renal failure and peripheral vascular disease). The performed descriptive statistics were: frequencies, percentages, means, and standard deviations, to find out the characteristics of the subject and the incidence and types of groin complications.

Data were subjected to inferential statistics, and two tailed *P* value of less than .05 was accepted to be statistically significant. To answer research question number one, descriptive statistics were used. For the second questions, to test the correlation between different patients’ characteristics and procedural techniques and the development of groin complications, Spearman rho and Pearson *r* coefficient tests were used according to the level of measurement for each variable; Pearson *r* test was used to assess the correlation between the continuous variables, and Spearman rho was used for the categorical variables. For the third question, to determine the most common predictors for the development of groin complications, logistic regression was used. Even though, logistic regression will give the results of odds ratio which might be amplified with respect to relative risks, we used it to control for other covariates that might affect the outcome of interest.

## Results

### Demographic and clinical characteristics

The sample included 237 (79%) males and 63 (21%) females with a mean age of 57.46 ± 10.51 years ranging from 31 to 82. Forty percent of the participants were less than 55 years old. The BMI mean was 28.96 ± 4.96. More than three quarters of the sample (78%) was overweight or obese. Regarding the clinical characteristics of the PCI patients, more than half of the sample had hypertension and 46.7% had diabetes mellitus. Sociodemographic and clinical characteristics of the sample are summarized in Table 2.

**Table 2:** Participant sociodemographic and clinical characteristics (*N* = 300)

Variable	Mean ± SD or n (%)
Age	57.46 ± 10.51
Less than 55 years	120 (40%)
Between 55 to 65 years	109 (36.3%)
More than 65 years	71 (23.7%)
BMI (Whole sample)	28.96 ± 4.96
Normal	66 (22%)
Overweight and obese	234 (78%)
BMI (Males)	28.23 ± 4.57
Normal	60 (25.3)
Overweight and obese	177 (74.7)
BMI (females)	31.71 ± 5.45
Normal	6 (9.5)
Overweight and obese	57 (90.5)
Comorbidities	
HTN	177 (59%)
DM	140 (46.7%)
HTN and DM	107 (35.7%)
PVD	5 (1.7%)
RF	3 (1%)
Gender	
Male	237 (79%)
Female	63 (21%)

*SD* Stander Deviation, *N* Number, *BMI* Body Mass Index, *HTN* Hypertension, *DM* Diabetes Mellitus, *PVD* Peripheral Vascular Disease, *RF* Renal Failure

Details of PCI procedure are summarized in Table 3. The mean of the heart rate when the procedure had started was 76 beats/minuet, (ranging from 45 to 112 beats/min). Most of the PCI procedures were performed through the right femoral artery (97.3%), with the majority completed using a 6 French sheath size (95%). The access site was penetrated mostly by one puncture to the femoral artery (80.3%).

**Table 3:** PCI procedure details (*N* = 300)

Variable	Mean ± SD or N (%)
Heart Rate	76.2 ± 11.6 beat/min
Site of Puncture	
Right groin	292 (97.3%)
Left groin	8 (2.7%)
Number of Punctures	
One punctures	241 (80.3%)
More Than One	59 (19.7%)
Location of Punctures	
Below inguinal crease	129 (43%)
On inguinal crease	155 (51.7%)
Above inguinal crease	16 (5.3%)
Coronary artery treated	
LAD	127 (42.3%)
CX	44 (14.7%)
RCA	61 (20.3%)
Others	68 (22.7%)
Time of Procedure	40.29 ± 18.4 Min
Sheath size	
5F	7 (2.3%)
6F	285 (95%)
7F	8 (2.7%)
Anticoagulant	
Heparin	10,563 ± 1302 IU
Glycoprotein Iib/IIIa	33 (11%)
Number of coronary treated	
One artery	235 (78.3%)
Two arteries	55 (18.3%)
Three arteries	10 (3.3%)
ACT	314 ± 34 Seconds
SBP when Procedure Started	150 ± 29 mmHg
Procedure performed	
PTCA	12 (4%)
PCI	288 (96%)

*PTCA* Percutaneous Transluminal Coronary Intervention, *PCI* Percutaneous Coronary Intervention, *LAD* Left Anterior Descending, *CX* Circumflex, *RCA* Right Coronary Artery, *F* French, *ACT* Activating Clotting Time at the end of the procedure

The majority of CAD patients were treated by stent implantation (PCI 96%), and 12 CAD patients were treated using only balloon inflation (PTCA 4%). Mostly, one coronary artery was in need for an intervention (78.3%), and the left anterior descending (LAD) artery was the highest frequently treated coronary artery (42.3%). The mean of invasive blood pressure when the procedure had started was 150 mmHg, ranging from 100 mmHg to 235 mmHg. Heparin as an anticoagulant was given for all treated patients in a mean dose of 10,563 IU, while Glycoprotein Iib/IIIa inhibitor was administered for 33 patients (11%).

Arterial sheath removal conditions are summarized in Table 4. The mean of ACT before sheath removal was 141.5 ± 15.4 s, and the mean time from the end of the procedure to the sheath removal (sheath duration) was 6.7 ± 1.4 h. One point 3 % of distal pulses, dorsalis pedis or posterior tibial, pre-sheath removal and post-sheath removal were not palpable. The mean of heart rate and systolic blood pressure at the time of sheath removal were 73 beats/min, and 132.2 ± 20.8 mmHg respectively. The mean of compression time was 10.5 ± 4.4 min.

**Table 4:** Arterial sheath removal conditions (*N* = 300)

Variable	Mean ± SD or N (%)
ACT before sheath removal	141.55 ± 15.43 S
Sheath duration	6.7 ± 1.4 Hours
Dorsalis pedis pulse pre-sheath removal	
Palpable	296 (98.7%)
Not Palpable	4 (1.3%)
Heart rate at time of sheath removal	73 ± 6.7 beat /min
SBP before sheath removal	132.4 ± 20.7 mmHg
Compression time	10.5 ± 4.4 Minutes
Dorsalis pedis pulse post sheath removal	
Palpable	296 (98.7%)
Not Palpable.	4 (1.3%)

*ACT* Activated clotting time, *SBP* Systolic blood pressure

### Research question number one

What is the incidence rate of groin complications post PCI procedure in primary cardiac catheterization laboratories services? A total of 114 patients (38%) developed one or more complications during hospitalization. The developed groin complications and their percentages are summarized in Table 5. The highest percentage was for two groin complications; ecchymosis was the most frequent groin complication 102 (34%), followed by small hematoma 60 (20%) then large hematoma 28 (9.3%). The least reported groin complication was arteriovenous fistula (0.3%). It is worthy to note that there was a remarkable drop in packed cell volume (PCV) in 24 patients (8%) of patients who developed bleeding and large hematoma. Neither the retroperitoneal bleeding nor arterial occlusion occurred in this study.

**Table 5:** Groin complications developed with their percentages (*N* = 300)

Variable	^a^n (%)
Groin complications	114 (38%)
Number of complications	
One complication	29 (9.7%)
Two complications	69 (23%)
Three complications	16 (5.3%)
Bleeding	9 (3%)
Hematoma	
Small hematoma	60 (20%)
Large hematoma	28 (9.3%)
Oozing	9 (3%)
Ecchymosis	102 (34%)
AV fistula	1 (0.3%)
Pseudoaneurysm	6 (2%)
Drop in PCV	24 (8%)

^a^ More than one patient developed more than one complication

### Research questions number two

Is there an association between socio- demographic characteristics (age, gender, BMI, diabetes mellitus, and hypertension) and groin complications post PCI procedure?

The correlation between socio-demographic characteristics (age, gender, BMI, diabetes mellitus, renal failure, and hypertension) and groin complications post PCI procedure are represented in Table 6. There was a positive significant relationship between age and the development of groin complications post PCI procedure. Moreover, there was a positive significant relationship between female gender, renal failure and the development of groin complications post PCI procedure. Female patients experienced an increased incidence of groin complications (*χ*^2^ = 10.43, *p* < .001), compared with male patients (55.6% vs 33.3%). The three renal failure patients who participated in this study developed groin complications. Other correlations were not statistically significant.

**Table 6:** Correlation between selected demographic and clinical characteristics and groin complications

Variables	Age	BMI	Gender	HTN	DM	RF	No. of punctures
Groin Complication	.177^*^	NS	.186^**^	NS	NS	.128^*^	NS

*Correlation is significant at *p* < 0.05. ** Correlation is significant at *p* < 0.005

*NS* Not Significant, *BMI* Body Mass Index, *HTN* Hypertension, *DM* Diabetes Miletus, *RF* Renal Failure, *No*. Number

### Research question number three

What are the most common predictors of groin complications post PCI procedure?

To investigate the predictors of groin complications post PCI procedure, two steps were done. First: a series of bivariate correlations between sociodemographic, clinical variables, procedure details, sheath removal conditions and the existence of groin complications were done. Then, the variables that were statistically significant at *p* < .1 were entered into a logistic regression model. We used *p* < .1 to be more conservative in the prediction.

The socio-demographic and clinical characteristics, procedural techniques and sheath removal conditions which were found to be statistically significant were age, gender, systolic blood pressure before sheath removal (SBPBSR), compression time, and ACT before sheath removal. The assumptions of multicollinearity was checked, and no violation was detected (Tolerance =0.642, VIF = 1.6).

The significant variables were entered to the logistic regression models to test their ability to predict groin complications. The variables were entered into three blocks. Demographic data including age categories (less than 55, between 55 and 65, and more than 65) and gender were entered in the first block. Comorbidities, including RF was entered in the second block. Finally, arterial sheath removal conditions including: ACT before sheath removal, categories of SBPBSR (below 140 mmHg, from 140 to 159 mmHg, from 160 to 180 mmHg, and more than180 mmHg) and compression time were entered in the third block.

When age was entered as a continuous variable it did not reach statistical significance as a predictor for complications development, which might be due to nonlinearity. Previous studies reported that age more than 65 years old was a predicting factor for groin complications development [14, 18, 24, 31–33]. For this reason, age was categorized into three categories (below 55 years, between 55 to 65 years, and more than 65 years). In addition, the systolic blood pressure more than 160 mmHg has been previously reported as a predicting factor for groin complications development [24, 34]. For this reason the SBPBSR was categorized in four categories (below 140 mmHg, from 140 to 159 mmHg, from 160 to 180 mmHg, and more than180 mmHg) [29].

The results showed that female gender, age more than 65 years old, and SBPBSR more than 180 mmHg were significant predictors of groin complications development Table 7. The SBPBSR more than 180 mmHg was a highly significant predictor for groin complications; when SBPBSR was greater than 180 mmHg, the patients were about ten times more likely to develop groin complications (OR = 9.82, *P* = .001, 95% CI: 2.58–37.37). Furthermore, patients whose age was more than 65 years old or were females were approximately double the time more likely to develop groin complications (OR = 2.13, *P* = .024, 95% CI: 1.11–4.01) and (OR = 2.14, *P* = .023, 95% CI: 1.11–4.13) compared to other groups.

**Table 7:** Predictors of groin complications

Predictor	OR	CI	B	Wald	*P*-value
Female gender	2.04	1.08–3.84	.712	4.83	.028
Age more than 65	2.13	1.11–4.10	.756	5.12	.024
SBPBSR more than 180 mmHg	9.82	2.58–37.37	2.28	11.23	.001

*OR* Odds Ratio. SBPBSR Systolic Blood pressure Before Sheath Removal. CI: 95% Confidence Interval

## Discussion

The major purpose of this study was to determine the incidence rate and predictors of groin complications early after PCI. The results showed that there were three predictors of complications (female gender, age above 65 years and SBPBSR more than 180 mmHg). The overall groin complication rate was 38% ranging from 0.3 to 38%. This rate is consistent with reported rates from previous related studies [10, 16, 17]. The previous incidence rates of groin complications have been reported to be anywhere from 0.1 to 61%, depending on the type of groin complication and the factors which were studied [10, 16, 17]. Sabo et al., (2008) reported that the overall occurrence of vascular complications post CCP range from 2 to 37% [18].

The majority of patients who developed groin complications had two forms of groin complications 23%, while 9.3% of the patients had only one form of groin complications. The reached rate for each groin complication was congruent with that reported in previous similar studies, except for the rate of oozing which was lower than previous studies (3%) [10, 12].

Some previous researches addressed groin oozing as a concern in comparing between the methods of hemostasis. Manual compression had the lowest oozing rate compared with other compression methods [15, 35]. Wu et al., (2015) concluded that the oozing is a mild vascular complication that can be resolved by reapplying compression over the puncture site and increasing the duration of bed rest [33]. The low rate of oozing obtained in the current study that was in match to previous studies, might be related to the hemostasis technique which was used. Manual compression followed by tight bandage dressing were used among all patients to obtain hemostasis, this technique has found to be associated with lower oozing rate [15, 35].

The most frequent groin complication was ecchymosis 34%. Groin ecchymosis may be considered a minor groin complication post CCP. However, the significance of ecchymosis should not be ignored, as reported in Cosman and colleague study [16], more than 4% of patients who developed ecchymosis post discharge sought for medical care related to groin pain and discomfort in the groin area [16]. Therefore, ecchymosis may have a significant implication in requesting patient follow ups.

The findings of this study are consistent with previous studies in identifying that female gender, age more than 65 years old, and SBPBSR more than 180 mmHg are significant predictors of groin complications development. The highest predictor in significance was SBPBSR more than 180 mmHg. This category was about ten times more likely to develop groin complications compared to other categories.

Dumont (2007) has previously reported that patients with systolic blood pressure during the procedure ≥160 mmHg were 8 times more likely to develop vascular complications compared to other groups [34]. Furthermore, Al Sadi et al., (2010) reported that the systolic blood pressure ≥ 160 mmHg measured at the beginning of the CCP is a significant predictor for groin hematoma development [24].

Kassem et al., (2013) revealed that patients with high blood pressure carry out a higher risk for the development of FAP, due to the difficulty of compressing an artery with elevated intra-luminal pressure [26]. Furthermore, all patients are advised to increase fluids intake (mostly water) post CCP, which elevates the preload of the heart. Also, many patients found it difficult to urinate while they are in complete bed rest. As a result, the blood pressure will be elevated, and the patients will become irritated. Moreover, high blood pressure against a weak point in the vascular structure will lead to bleeding complications.

On the other hand, Konstance et al., (2004) reported that the inverse relationship between hypertension and major vascular complications with no clear explanation, and this inverse relationship has not been previously reported [32]. Furthermore, Dumont (2007) reported that chronic hypertensive patients were 60% less likely to have vascular complications [34]. However, this result might be explained as the following: blood vessels of patients with chronic hypertension are more adapted to increased blood pressure reading than those who are newly diagnosed with high blood pressure due to a recent cardiac event. More studies to check the effect of hypertension on complication development are still needed.

This study showed that patients older than 65 years were at approximately two times higher risk for developing complications than other age groups. Wu et al., (2015) reported that patients older than 70 years were 10.44 times more likely to devolve groin complications than patients aged less than 70 years [33]. Moreover, Konstance et al., (2004) concluded that an each year advance in age increases the probability of groin complication development by 5% [32].

The reduction in muscles mass, decrease in elasticity of arterial wall, atherosclerosis and high calcified femoral artery, and the slow healing process in the elderly are factors that could affect the hemostasis process [14, 18]. However, Eisen et al., (2013) reported that age more than 60 years old and males have a protective effect against retroperitoneal bleeding by 70% without a clear explanation [31].

The current study showed that females were two times more likely to develop groin complications than male patients. It was reported that females were at higher risk for developing groin complications compared to males by 84% [26]. Moreover, Konstance et al., (2004) reported that the females were triple the times more likely to develop groin complications compared to males [32]. Furthermore, Lichtman et al., (2014) showed that females are more likely to develop groin bleeding complications compared to males [25].

Lichtman and colleagues explained that hormone levels such as estrogen may modulate endothelial functions by increasing the level of coagulation factors and inflammatory markers [25]. The structure of the female body has differences compared to males which might increase the susceptibility for complication development. These might include but are not limited to: less muscle mass, less vessel diameter and more adipose tissue. Muscle mass helps in raising intrinsic compression which supports the punctured artery. Furthermore, females have smaller vessel sizes than males, which may increase procedural complexity and the risk of residual vascular injury [25, 31].

## Conclusion

Different factors can increase the risk of groin complications development post PCI procedure. Health care providers have a major role and responsibility to know and to detect such complications. The findings of the current study can be used by health care providers to prevent or minimize the incidence of groin complications and prevent its consequences post PCI procedure by considering high risk groups (i.e. females and hypertensives).

### Limitations

The major limitation of this study is the timing of the data collection. Data for the current study was collected through a 24 h follow up post PCI procedure, and any groin complication experienced within this time was recorded. But any groin complication developed after that time has gone undetected. Moreover, the data was collected from one specialized Cardiac Health Care Center. Even though Cardiac Health Care Center provides health care for a widespread of cardiac diseases patients in Jordan and other countries, there are other centers in Jordan which can be included in further studies.

### Recommendation for future studies

New studies including different setting of the health care sectors and higher numbers of females are recommended. Further studies checking the effect of hypertension on complication are still necessary.

## Data Availability

All data generated or analyzed during this study are included in this published article and its supplementary information files.

## Abbreviations

ACT: Activating clotting time
AVF: Arteriovenous fistula
BMI: Body mass index
CAD: Coronary artery disease
CCP: Cardiac catheterization procedure
CHCC: Cardiac Health Care Center
CI: Confidence interval
CVD: Cardiovascular disease
CX: Circumflex
F: French
FAP: Femoral artery pseudoaneurysm
LAD: Left anterior descending
OR: Odds ratio
PCI: Percutaneous coronary intervention
PTCA: Percutaneous transluminal coronary intervention
RCA: Right coronary artery
SBP: Systolic blood pressure
SBPBSR: Systolic blood pressure before sheath removal
SD: Standerd deviation
SPSS: Statistical package for social sciences

## References

[1] Benjamin EJ, Blaha MJ, Chiuve SE, Cushman M, Das SR, Deo R (2017). Heart disease and stroke statistics—2017 update: a report from the American Heart Association. Circulation..

[2] Aljefree Najlaa, Ahmed Faruk (2015). Prevalence of Cardiovascular Disease and Associated Risk Factors among Adult Population in the Gulf Region: A Systematic Review. Advances in Public Health.

[3] Ohira T, Iso H (2013). Cardiovascular disease epidemiology in Asia. Circ J.

[4] Wilkins E, Wilson L, Wickramasinghe K, Bhatnagar P, Leal J, Luengo-Fernandez R (2017). European cardiovascular disease statistics 2017.

[5] Bell Beth P., Damon Inger K., Jernigan Daniel B., Kenyon Thomas A., Nichol Stuart T., O’Connor John P., Tappero Jordan W. (2016). Overview, Control Strategies, and Lessons Learned in the CDC Response to the 2014–2016 Ebola Epidemic. MMWR Supplements.

[6] Lee GA (2009). Determinants of quality of life five years after coronary artery bypass graft surgery. Heart Lung.

[7] Best PJ, Lennon R, Gersh BJ, Ting HH, Rihal CS, Bell MR (2003). Safety of abciximab in patients with chronic renal insufficiency who are undergoing percutaneous coronary interventions. Am Heart J.

[8] Kushner FG, Hand M, Smith SC, King SB, Anderson JL, Antman EM (2009). Focused updates: ACC/AHA guidelines for the Management of Patients with ST-elevation myocardial infarction (updating the 2004 guideline and 2007 focused update) and ACC/AHA/SCAI guidelines on percutaneous coronary intervention (updating the 2005 guideline and 2007 focused update). Catheter Cardiovasc Interv.

[9] Agostoni P, Anselmi M, Gasparini G, Morando G, Tosi P, De Benedictis ML (2006). Safety of percutaneous left heart catheterization directly performed by cardiology fellows: a cohort analysis. J Invasive Cardiol.

[10] Bashore TM, Bates ER, Berger PB, Clark DA, Cusma JT, Dehmer GJ (2001). American College of Cardiology/Society for Cardiac Angiography and Interventions Clinical Expert Consensus Document on cardiac catheterization laboratory standards: a report of the American College of Cardiology Task Force on clinical expert consensus documents endorsed by the American Heart Association and the diagnostic and interventional catheterization Committee of the Council on clinical cardiology of the AHA. J Am Coll Cardiol.

[11] Dumont CJ, Keeling AW, Bourguignon C, Sarembock IJ, Turner M (2006). Predictors of vascular complications post diagnostic cardiac catheterization and percutaneous coronary interventions. Dimens Crit Care Nurs.

[12] Kern MJ, Lim MJ, Sorajja P. The interventional cardiac catheterization handbook E-book: Elsevier Health Sciences; 2017.

[13] Smith SC, Feldman TE, Hirshfeld JW, Jacobs AK, Kern MJ, King SB (2006). ACC/AHA/SCAI 2005 guideline update for percutaneous coronary intervention: a report of the American College of Cardiology/American Heart Association task force on practice guidelines (ACC/AHA/SCAI writing committee to update the 2001 guidelines for percutaneous coronary intervention). J Am Coll Cardiol.

[14] Badr S, Kitabata H, Torguson R, Chen F, Suddath WO, Satler LF (2014). Incidence and correlates in the development of iatrogenic femoral pseudoaneurysm after percutaneous coronary interventions. J Interv Cardiol.

[15] Chlan LL, Sabo J, Savik K (2005). Effects of three groin compression methods on patient discomfort, distress, and vascular complications following a percutaneous coronary intervention procedure. Nurs Res.

[16] Cosman TL, Arthur HM, Natarajan MK (2011). Prevalence of bruising at the vascular access site one week after elective cardiac catheterisation or percutaneous coronary intervention. J Clin Nurs.

[17] Merriweather N, Sulzbach-Hoke LM (2012). Managing risk of complications at femoral vascular access sites in percutaneous coronary intervention. Crit Care Nurse.

[18] Sabo J, Chlan LL, Savik K (2008). Relationships among patient characteristics, comorbidities, and vascular complications post-percutaneous coronary intervention. Heart Lung.

[19] Ahn H-Y, Lee H-J, Lee H-J, Yang J-H, Yi J-S, Lee I-W (2014). Assessment of the optimal site of femoral artery puncture and angiographic anatomical study of the common femoral artery. J Korean Neurosurg Soc.

[20] Fairley SL, Lucking AJ, McEntegart M, Shaukat A, Smith D, Chase A (2016). Routine use of fluoroscopic-guided femoral arterial puncture to minimise vascular complication rates in CTO intervention: multi-centre UK experience. Heart Lung Circ.

[21] Kalapatapu VR, Ali AT, Masroor F, Moursi MM, Eidt JF (2006). Techniques for managing complications of arterial closure devices. Vasc Endovasc Surg.

[22] Sobolev M, Slovut DP, Lee AC, Shiloh AL, Eisen LA. Ultrasound-Guided Catheterization of the Femoral Artery: A Systematic Review and Meta-Analysis of Randomized Controlled Trials. 2015. 26136279

[23] Van Den Berg J. Optimal technique for common femoral artery access. Endovascular today. 2013:58–61.

[24] Al Sadi AKA, Omeish AFY, Al-Zaru IM (2010). Timing and predictors of femoral haematoma development after manual compression of femoral access sites. JPMA J Pakistan Med Assoc.

[25] Lichtman JH, Wang Y, Jones SB, Leifheit-Limson EC, Shaw LJ, Vaccarino V (2014). Age and sex differences in inhospital complication rates and mortality after percutaneous coronary intervention procedures: evidence from the NCDR®. Am Heart J.

[26] Kassem HH, Elmahdy MF, Ewis EB, Mahdy SG (2013). Incidence and predictors of post-catheterization femoral artery pseudoaneurysms. Egyptian Heart J.

[27] Erol F, Arslan Ş, Yüksel İÖ, Üreyen ÇM, Serdar S, İnci S (2015). Determinants of iatrogenic femoral pseudoaneurysm after cardiac catheterization or percutaneous coronary intervention via the femoral artery. Turk Kardiyol Dern Ars.

[28] Al-Makhamreh HK, Alqsous N, Ammari Z, Husari K, Nimri S, Ya’aqoub L, et al. Vascular complications following cardiac catheterization at Jordan University hospital. Jordan Med J. 2014;48(4).

[29] Bickley L, Szilagyi PG. Bates' guide to physical examination and history-taking: Lippincott Williams & Wilkins; 2012.

[30] Polit DF, Beck CT. Nursing research: generating and assessing evidence for nursing practice: Lippincott Williams & Wilkins; 2008.

[31] Eisen A, Kornowski R, Vaduganathan M, Lev E, Vaknin-Assa H, Bental T (2013). Retroperitoneal bleeding after cardiac catheterization: a 7-year descriptive single-center experience. Cardiology..

[32] Konstance R, Tcheng JE, Wightman MB, Kelly LP, Moore A, Harrison JK (2004). Incidence and predictors of major vascular complications after percutaneous coronary intervention in the glycoprotein IIb/IIIa platelet inhibitor era. J Interv Cardiol.

[33] Wu P-J, Dai Y-T, Kao H-L, Chang C-H, Lou M-F (2015). Access site complications following transfemoral coronary procedures: comparison between traditional compression and angioseal vascular closure devices for haemostasis. BMC Cardiovasc Disord.

[34] Dumont CJ (2007). Blood pressure and risks of vascular complications after percutaneous coronary intervention. Dimens Crit Care Nurs.

[35] Mohammed H, Said H, Salah M (2013). Determining Best nursing practice: effectiveness of three groin compression methods following cardiac catheterization. J Am Sci.

